# Microrobotic Platform for Single Motile Microorganism Investigation

**DOI:** 10.3390/mi8100295

**Published:** 2017-09-30

**Authors:** Belal Ahmad, Hironobu Maeda, Tomohiro Kawahara, Fumihito Arai

**Affiliations:** 1 Graduate School of Life Science and Systems Engineering, Kyushu Institute of Technology, Kitakyushu 808-0196, Japan; ahmad-belal-bashir@edu.life.kyutech.ac.jp; 2 School of Engineering, Kyushu Institute of Technology, Kitakyushu 804-8550, Japan; o108105h@mail.kyutech.jp; 3 Graduate School of Engineering, Nagoya University, Nagoya 464-8603, Japan; arai@mech.nagoya-u.ac.jp

**Keywords:** automated microscopy, target tracking, motile microorganism, visual servoing

## Abstract

We propose a microrobotic platform for single motile microorganism observation and investigation. The platform utilizes a high-speed online vision sensor to realize real-time observation of a microorganism under a microscopic environment with a relatively high magnification ratio. A microfluidic chip was used to limit the vertical movement of the microorganism and reduce the tracking system complexity. We introduce a simple image processing method, which utilizes high-speed online vision characteristics and shows robustness against image noise to increase the overall tracking performance with low computational time consumption. The design also considers the future integration of a stimulation system using microtools. Successful long-time tracking of a freely swimming microorganism inside of a microfluidic chip for more than 30 min was achieved notwithstanding the presence of noises in the environment of the cell. The specific design of the platform, particularly the tracking system, is described, and the performance is evaluated and confirmed through basic experiments. The potential of the platform to apply mechanical stimulation to a freely swimming microorganism is demonstrated by using a 50-µm-thick microtool. The proposed platform can be used for long-term observation and to achieve different kinds of stimulations, which can induce new behavior of the cells and lead to unprecedented discoveries in biological fields.

## 1. Introduction

Investigation of single motile microorganisms has become one of the most important topics in biology. It is known to have high potential in the fields of food production [1], bio-fuel, and neurology [2], and related research seeks to understand the specific function of mechanoreceptors and the growth factor of microorganisms. In addition, the motility function of microorganisms, such as bacteria, has been actively utilized for delivering drugs to targeted organs in state-of-the-art nanomedicine applications [3]. To clarify the characteristics and functions of microorganisms, the stimulus response of microorganisms is examined by applying external stimulations [4,5,6,7]. External stimulation and its response can give us unique information about cell characteristics [8,9,10,11]. For example, *Paramecium* electrical stimulation has been employed on a single-cell level to train the organism in a discrimination-learning task [12]. Because many aquatic microorganisms have motility and agility, it is quite difficult to investigate their dynamic behavior and stimulus response under a microscopic environment. Therefore, to conduct observations in a highly magnified environment, the motile microorganisms are immobilized before the investigation to drastically reduce their activity [13]. However, this immobilization acts as an external force that affects the functionality and response of the microorganism. To obtain exact natural behavior, external influences on aquatic microorganisms (except the applied stimulus) should be avoided as much as possible while they are moving in a fluidic environment.

On the other hand, freely swimming microorganisms can easily move outside of the field of view (FOV) of a microscope. In fact, when a normal camera with a frame rate of 30 frames per second (FPS) is used, it is impossible to observe the specific behavior and response of the microorganism for a long time. This means that observing relatively fast objects under a microscope with a vision sensor is closely related to the vision frame rate, and using a larger magnification ratio requires higher frame rate vision sensors. To solve this problem, a high-speed online vision sensor has been introduced for the real-time observation of motile microorganisms. In particular, Ogawa et al. developed a high-speed tracking system composed of a high-speed online vision sensor and motorized stages [14]. By using the visual servoing technique, they succeeded in the continuous tracking of single *Paramecium* under the microscope, even in three-dimensional (3D) space. However, in their system, the magnification ratio was fixed during tracking, and the maximum tracking time was at the 60-s level, although it is highly desirable to observe the target cell for a longer time. Furthermore, local point stimulation of a single motile microorganism with free swimming has not been achieved. It is worth mentioning that microorganism tracking has been achieved using fiber optics and analog comparators in the pioneer works [15,16].

**Figure 1:**
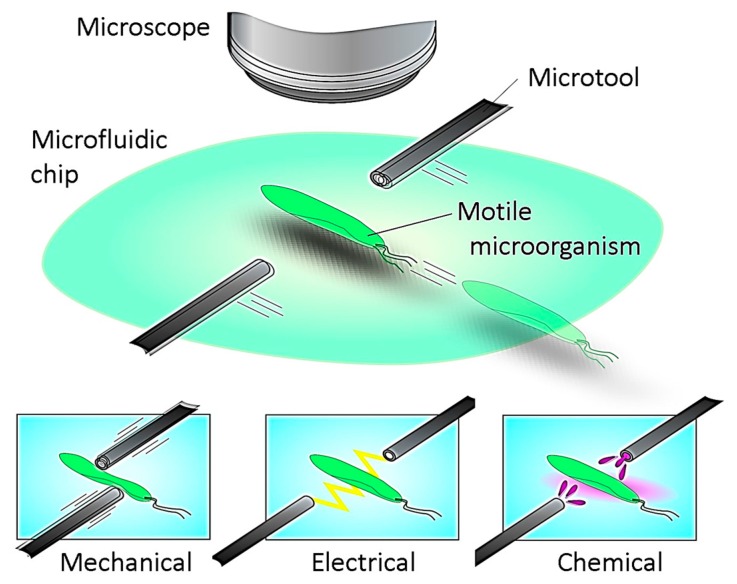
Conceptual image of proposed microrobotic platform. Motile microorganisms are inserted into a microfluidic chip and continuously tracked by a microscope equipped with a high-speed online vision sensor for a long time. External stimulus is then locally applied by using microtools to investigate the stimulus response characteristics of a single motile microorganism.

Based on the background described above, we newly propose a microrobotic platform that can realize both continuous observation and local point stimulation for a freely swimming, single microorganism in a microfluidic chip. The platform will allow us to apply different kinds of stimulations to a single cell and observe its nearly natural response without immobilization, as shown in Figure 1. Our target is motile microorganisms with a size of several hundred micrometers and a maximum speed of several millimeters per second. In this paper, basic design and implementation of the platform to satisfy the required specifications are shown. An adaptive visual servoing technique to maintain the target cell in the center of the FOV over a long period of time is then introduced and examined. Finally, we apply the developed platform for actual motile cell tracking and stimulation experiments.

## 2. Microrobotic Platform

### 2.1. Required Specifications

Motile microorganisms move within an aquatic environment in three dimensions (3D), which makes it considerably difficult to continuously observe the target microorganism. Reducing the movement of the cell to only 2D would drastically simplify both the observation and stimulation processes. This can be achieved using a thin microfluidic chip that bounds the movement of the cell in the vertical plane. It is worth mentioning that the moving directions of the motile cell could be further decreased from 2D to 1D by introducing the cell into a flow path with a microchannel on a microchip. However, in the case of a motile microorganism, much labor is required to induce it to the desired position by fluidic control. Therefore, a microfluidic chip with a flat space is utilized for our platform. The thickness of the flat space can be precisely adjusted by using a microfabrication technique.

It is relatively easy to observe the position and movement of a swimming microorganism in a low-magnification environment. However, a high magnification ratio is preferred to observe specific behavior and reactions. Consequently, the relative speed of microorganisms increases with an increasing magnification ratio because the time duration of the microorganisms in the observation FOV decreases. Therefore, tracking the position of a single microorganism continuously is quite difficult. Therefore, to best obtain observations with a high magnification ratio, together with a high spatial resolution, a high-speed online vision sensor is required. In addition, it is difficult to conduct the experiment starting immediately from a highly magnified environment, especially in the searching process, which is a laborious task. Therefore, a seamless magnification changing mechanism is also needed to improve the efficiency of the experiment.

On the other hand, because our platform is designed to apply to real-time stimulation, the stimulation tool might reduce the tracking stability, especially when the tool is in direct contact with the target cell (e.g., mechanical stimulation). Moreover, the microorganism behavior must be observed after applying the stimulation to confirm the effect of the stimulation on the cell. Therefore, to build an effective tracking system, we must recognize the position of the target regardless of other objects visible in the FOV that we consider as noise. In fact, it is possible to eliminate the image noises using conventional image processing algorithms for object detection and classification [17,18]. Nonetheless, we must use a high-speed online vision sensor, which implies that the processing time for each frame is small and thus that it would be difficult to use time-consuming detection algorithms. Hence, a maximally simplified, robust image processing technique is required.

### 2.2. System Components

**Figure 2:**
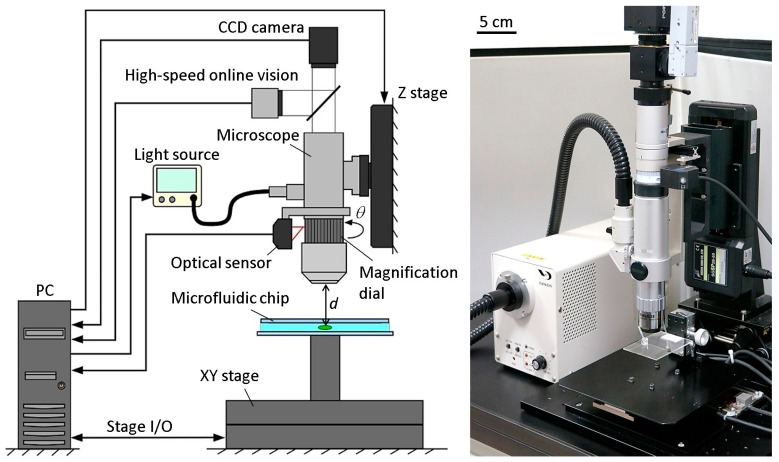
Main components of the developed platform. Windows 7 64-bit operating system (OS) was installed on a PC with 32 GB of physical memory. It was equipped with 16-bit AD/DA converter boards, 32-bit counter boards, and an FPGA board for a high-speed online vision sensor. *d* is the distance between the microscope lens and the target microorganism (the focal length), and *θ* is the rotational angle of the magnification dial. The relationship between the magnification ratio and the spatial resolution of the FOV for the high-speed online vision is as follows: [140× 210×, 280×, 420×, 560×, 700×, 840×, 980×, 1120×, 1400×] = [5.50, 4.80, 3.50, 2.20, 1.66, 1.29, 1.09, 0.92, 0.85, 0.79][µm/pixel]. The CCD camera’s FOV dimensions ranged from 1907 µm × 1073 µm to 231 µm × 130 µm for magnification ratios ranging from 140× to 1400×.

Figure 2 shows the basic components of the developed microrobotic platform. A high-speed online vision sensor (IDP-ExpressR2000, Photron Inc., Tokyo, Japan) [19] was implemented into the microscope for high-speed image capturing. The vision sensor’s parameter of 256 × 256 pixels at 500 FPS was used for all of the experiments in this paper. A CCD camera (ZBR2-PGEHD-28S4C-CS, Point Grey Research Inc., Now FLIR Integrated Imaging Solutions Inc., Richmond, BC, Canada) was also attached to the microscope for observing the behavior of the microorganism. The CCD camera can capture color images of 1920 × 1080 pixels at 15 FPS. A 140× lens was installed into the digital microscope (DVM2000, Leica Microsystems Inc., Buffalo Grove, IL, USA), and the magnification ratio could be seamlessly changed from 140× to 1400× by rotating the magnification dial attached to the lens barrel of the microscope. A metal scale was attached to the magnification dial, along with an optical sensor (VP-90, Keyence Inc., Osaka, Japan) to detect the dial’s position. The microscope was mounted onto a Z stage (SGSP 26-50, Sigma Koki Inc., Tokyo, Japan), which was controlled by the PC to maintain the focal length when changing the magnification ratio. The Z stage was driven by a stepping motor with a positioning accuracy of 3 µm and a maximum drive speed of 30 mm/s. Depending on the change of the magnification ratio, the light source (LA-HDF 5010, Hayashi Watch-Works Inc., Tokyo, Japan) was also adjusted to maintain the consistency of the light intensity. The specific control method for the magnification ratio of the microscope will be detailed in Section 2.3.

By obtaining the target position data from the vision sensor, an XY stage (SGTMM03-065AH20A, Yasukawa Electric Inc., Kitakyushu, Japan) was controlled by the PC to maintain the microorganism in the FOV of the microscope. It was driven by a linear AC motor with a positioning accuracy of 200 nm and maximum drive speed of 1500 mm/s. Simple adaptive control [20] was used for controlling the XY stage to compensate for the parameter variations of the system, such as friction and nonlinearity. The visual tracking algorithm of a single motile microorganism will be shown in Section 2.4.

A microfluidic chip, made of a glass-PDMS-glass sandwich, was designed in a way that seals all of the edges except for one, which was unsealed to allow for the extremal insertion of microtools. The microchip had no liquid leakage, even while actuating the tool, thanks to the high water tension between the two glass substrates. The microchip was mounted onto the XY stage directly. The workspace in the microchip was 30 mm × 30 mm in length and width, respectively. The appropriate thickness of the microchip for actual motile microorganisms will be discussed in the experimental section. 

### 2.3. Magnification Ratio Control of Microscope

To improve the efficiency of the investigations, it is better to start the visual tracking from a low magnification ratio to quickly find a target microorganism. The magnification ratio is then changed seamlessly to a high magnification for obtaining high-resolution images. While changing the magnification ratio, the target tracking control should be stably maintained. To realize this function, three main factors that affect the tracking control continuity should be considered. These three parameters are the pixel pitch *p* (i.e., the distance from the center of a pixel to the center of the next pixel measured in millimeters)*,* the focal length *d*, and the light intensity *l*, where only the pixel pitch has a direct effect on the tracking control. We assume that the magnification ratio can be changed without replacing the lens through manually rotating a magnification dial on the microscope to zoom in and out. Because the magnification dial rotational degree *θ* indicates the currently applied magnification ratio, each of the parameters can be mathematically modelled as a function of *θ* as follows:

(1) $$\left[\begin{array}{c} p\\ d\\ l \end{array}\right]=\left[\begin{array}{c} f_{1}\left(\theta \right)\\ f_{2}\left(\theta \right)\\ f_{3}\left(\theta \right) \end{array}\right]$$

Therefore, real-time control of the three parameters would be achievable, allowing for a seamless change of the magnification ratio without affecting the tracking stability. The modelling functions were obtained by a calibration process for each parameter. To realize this method, at each calibration point, the pixel pitch was measured, and both the focal distance and the light intensity were adjusted and measured. At the same time, the rotational degree at each calibration point was measured using an optical rotation sensor in real time. Using the calibration data, the mathematical models of the three parameters were obtained by fitting curves. The fitting curves for three parameters, along with the curve equations are shown in Figure S1. To seamlessly change the magnification ratio, the optical sensor attached to the microscope measures the rotational degree of the magnification dial and sends this information to the controller, where the three parameters (*p*, *d*, and *l*) are calculated using the curve equations. The controller then sends commands to each of the Z stage and the light source to adjust both the focal distance and the light intensity. The pixel pitch is also adjusted simultaneously. The effectiveness of the developed real-time magnification ratio control can be confirmed from Video S1. Furthermore, the efficiency of this method will be confirmed through basic experiment in Section 3.

### 2.4. Visual Tracking Algorithm

**Figure 3:**
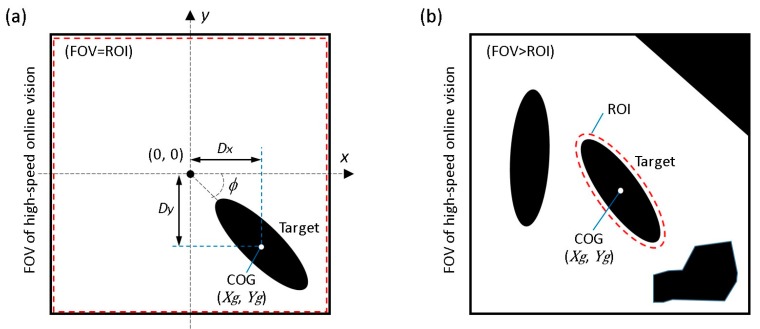
Image coordinate system for image processing. (**a**) Typical image processing for target tracking; (**b**) Implemented approach with an elliptical region of interest (ROI).

The objective of the visual tracking is to maintain the target motile microorganism in the center of the microscope’s FOV using a high-speed visual servoing technique [14]. The vision sensor detects the position of the microorganism and feeds this information back to the XY stage to maintain the target in the center of the FOV. To detect the target’s position, a grayscale image captured by the vision sensor is converted into a binary image. In our algorithm, an adaptive thresholding technique [21] is used to adaptively change the threshold value to compensate for illumination variations in real time. The center of gravity (COG) of the target ($X_{g},\;Y_{g}$), as shown in Figure 3a, can then be calculated from the 0th- and 1st-order moments of the binary image as follows:

(2) $$M_{i,j}=\underset{x}{\sum }\underset{y}{\sum }x^{i}y^{j}I\left(x,y\right)$$

(3) $$X_{g}=\frac{M_{1,0}}{M_{0,0}}$$

(4) $$Y_{g}=\frac{M_{0,1}}{M_{0,0}}$$

where $M_{i,j}$ represents the *i,j*-order image moment, $X_{g}$, $Y_{g}$ represent the image COG coordinates, and I(x,y) represents the pixel intensity at the index (*x*, *y*). In addition, the posture of the target ϕ can be obtained from the 2nd-order moment,

(5) $$\phi =\frac{1}{2}\tan^{-1}\left(\frac{2M_{1,1}}{M_{2,0}-M_{0,2}}\right)$$

**Figure 4:**
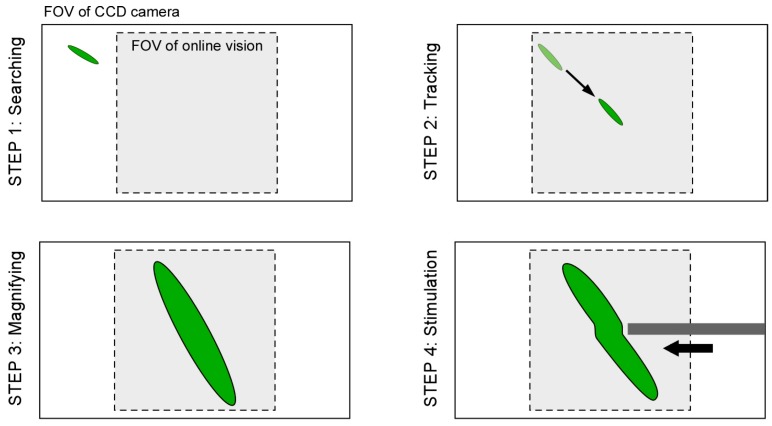
Four-step procedure to realize investigation of single motile microorganism.

Here, we suppose two main assumptions as follows; (i) because 3D movement of motile microorganisms is limited by a microfluidic chip, there is no overlap between each microorganism (no occlusion) during the observation; (ii) because the high-speed online vision sensor is used, the difference between each image frame is very small. Based on the assumptions above, specific procedures of the proposed visual tracking algorithm of a microorganism are shown in Figure 4.

**Step 1:** At first, by using the lowest magnification ratio of the microscope, the XY stage is moved with constant speed to find a microorganism as quickly as possible.

**Step 2:** If the vision finds the target (the total number of black pixels exceeds the threshold value), the visual tracking is started automatically. The XY stage is controlled such that the target’s position comes to the FOV center of the vision (*x*, *y*) = (0, 0).

**Step 3:** During the visual tracking, the magnification ratio of the microscope is seamlessly changed to obtain the enlarged view of the target by manually rotating the magnification dial. While increasing the magnification ratio, the pixel pitch, focal point, and light intensity are controlled in real time, as described in Section 2.3. Therefore, the visual tracking is maintained continuously.

**Step 4:** During the tracking with the high magnification ratio of the microscope, local stimulation of target is conducted by inserting a microtool from the side space of the microfluidic chip. The specific behavior of the target is then recorded by using the high-resolution CCD camera.

However, because there are many objects that can cause image noises in actual environments, it is quite difficult to track the target for a long time because the wrong position of the target COG is obtained. As a result, the visual tracking fails immediately, especially in the case of a highly magnified environment. In fact, a microfluidic chip includes significant debris and bubbles together with motile microorganisms, and the edge of the chip recognized as a darker area should be considered as image noise. Furthermore, when we attempt to apply stimulation to the target using the microtools, the calculated COG is moved from the target to the microtool, and the platform tracks the tool. This means that we cannot continue the observation of the target after the stimulation process. Although many effective algorithms to eliminate surrounding noise have been proposed and implemented in the field of image processing, the computing cost of their approaches is relatively high. To realize a high-speed tracking system, many images must be processed in real time, and the control thread must be maintained in the order of milliseconds by using a simple algorithm.

To overcome this dilemma, we introduce a simple visual tracking algorithm with high robustness by actively considering the characteristic of a high-speed online vision sensor. As described above, the difference between each frame is very small in high-frame-rate image capture, which contributes to simplify the image processing algorithm. In STEP 3, under the stable tracking and highly magnified condition, the region of interest (ROI) for image processing is shrunk to only around the target as shown in Figure 3b. Here, the shape of the ROI at the *k*-th image frame is determined as follows:

(6) $${\mathrm{ROI}}^{k}:\;\frac{{\left\{\left(x-X_{g}^{k-1}\right)\cos\phi^{k-1}+\left(y-Y_{g}^{k-1}\right)\sin\phi^{k-1}\right\}}^{2}}{a^{2}}+\frac{{\left\{-\left(x-X_{g}^{k-1}\right)\sin\phi^{k-1}+\left(y-Y_{g}^{k-1}\right)\cos\phi^{k-1}\right\}}^{2}}{b^{2}}=1$$

where *k −* 1 means the target’s information in the previous frame, and *a* and *b* are the semimajor axis and the semiminor axis of the ellipse, respectively. Therefore, by using the previous frame’s information (COG and posture of the target) to determine the ROI for the current frame, we can reduce surrounding image noise and processing cost simultaneously. Although similar techniques have been used in other works with high-speed vision systems [22,23,24], these systems did not have a magnification changing mechanism and did not focus on long-time target tracking.

**Figure 5:**
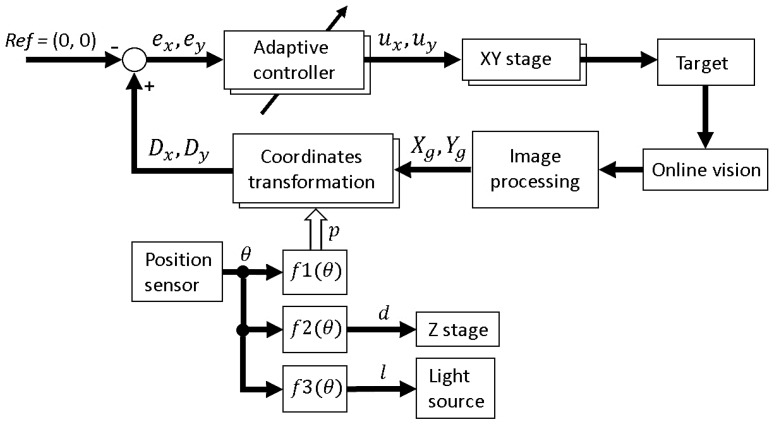
Block diagram for the developed microrobotic platform.

The complete block diagram of the platform is shown in Figure 5. In the next section, the robustness of the implemented visual tracking algorithm with the magnification ratio control function is evaluated experimentally and applied to an actual motile microorganism. In these experiments, we are interested in testing the tracking system stability in the case of a noisy environment rather than the accuracy of tracking. This means that the tracking is considered successful if the tracking system is able to continuously track the target, even with a relatively large tracking error.

## 3. Experiments

### 3.1. Magnification Ratio Control Efficiency

**Figure 6:**
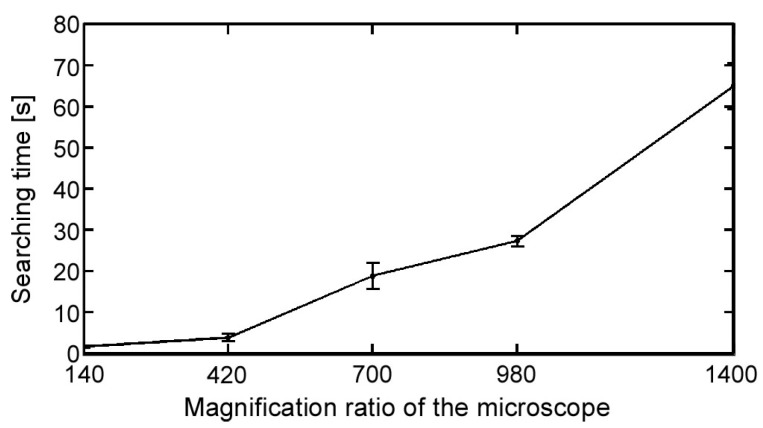
Magnification ratio control efficiency in reducing the searching time required to find a target. The field of view (FOV) dimensions of the online vision ranged from 1408 µm × 1408 µm to 202 µm × 202 µm.

To clarify the efficiency of the magnification ratio control implemented in our platform, we have confirmed the time required to search for a target. In this experiment, a number of polystyrene beads were scattered on a glass substrate mounted under the microscope to simulate actual targets. The beads were scattered within a 10 mm × 10 mm square around the microscope’s FOV. The XY stage was then moved in a Lissajous curve trajectory to find a target bead and the searching was terminated judging by the target area (the number of black pixels in the binary image). The searching experiment was repeated using a number of magnification ratios available in the platform, and the time required for each case was recorded. A total of five searching experiments were done for each magnification ratio and the mean value for each case was calculated. The polystyrene beads were all steady and had the same position with respect to the FOV in all cases to have a fair comparison. Figure 6 shows the searching time required for each magnification ratio. As we can see from the figure, it is clear that using a lower magnification ratio drastically reduces the required searching time, and hence reducing the total experimental time, which increases the efficiency and repeatability of the experiment.

### 3.2. Tracking Performance Evaluation

**Figure 7:**
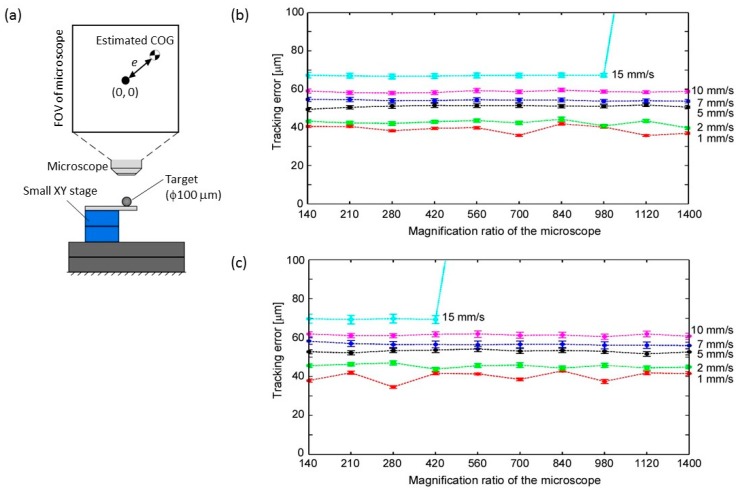
Tracking performance evaluation of developed platform. (**a**) Experimental setup; (**b**) Tracking error with typical image processing method; and, (**c**) Tracking error with implemented image processing method.

First, we examined the maximum tracking performance of the developed platform under the ideal (noiseless) condition. In this experiment, another small XY stage was mounted to simulate the movement of the motile cell as shown in Figure 7a. A polystyrene bead with a diameter of 100 µm was attached to the tip of the stage under the microscope as a tracking target and moved in a circular trajectory (φ2.0 mm) at different moving velocities. The target was then tracked to the center of the microscope’s FOV under the fixed magnification ratio (Video S2). The tracking error, which is the distance between the center of the FOV and the target position, as shown in Figure 7a, was calculated (each plotted point in Figure 7b,c is the mean value of 1000 samples, and the error bars show the standard deviation of each 1000 samples). 

Regarding the image processing, two cases were demonstrated. In the first case, the entire FOV of the microscope was considered as the ROI for the image processing (Figure 7b). In the second case, a selected ROI as explained in Section 2.4 was implemented (*a* = *b* = 20 in Equation (6), circle ROI) and performed (Figure 7c). The ROI parameters where determined based on the size of the target, where in this case the ROI is slightly larger than the polystyrene bead size when viewed under 140× magnification. In both cases, the computational cost of the image processing was within 3 ms.

From these results, it was confirmed that the tracking error increases with increasing target velocities, and the tracking failed at relatively large magnification ratios with target velocity greater than 15 mm/s. On the other hand, at target velocity less than 10 mm/s, the tracking performance was maintained in both cases even if the magnification ratio reached 1400×. Through the experiment, we confirmed the limitation of the target tracking on the developed platform. In the case of a noiseless environment, there was small difference of the tracking performance between the two cases of the image processing method.

**Figure 8:**
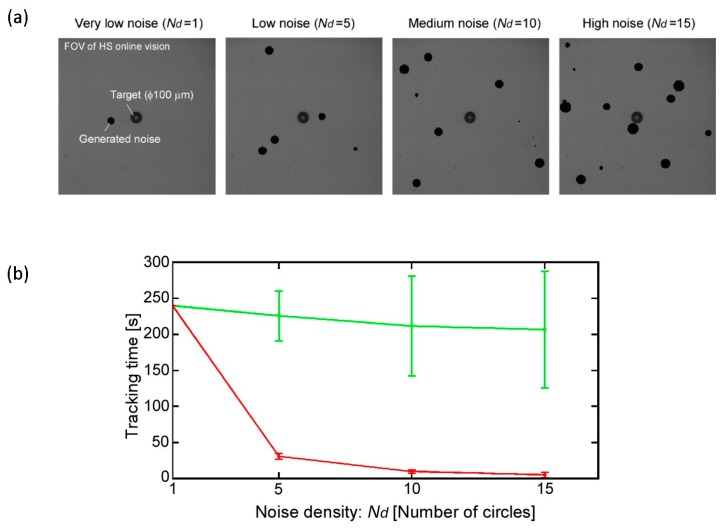
Robustness evaluation of target tracking in noisy environment. (**a**) Examples of four different noisy situations; (**b**) Total tracking time with typical image processing method (red) and implemented image processing method (green).

Next, we evaluated the robustness of the implemented tracking method. We simulated a noisy image situation by adding software-generated noise to the captured image in each frame. The generated noise has a circular shape with image coordinates and size that are generated and updated randomly in each frame. In this experiment, the assessment was based on the duration of tracking with the magnification ratio of 140× (Video S2). Examples of the noisy images are shown in Figure 8a. Four different noisy situations were tested, each of which had different amounts of generated noise circles. The four situations were very low noise, low noise, medium noise, and high noise density, where the numbers of circles in each situation were 1, 5, 10, and 15, respectively. It is important to note that the noise was restricted from being generated in coordinates that caused it to overlap with the target because this situation is outside the scope of this work.

Figure 8b shows the experimental result. For the image processing, two cases were demonstrated with the same conditions as the above experiments. A total of six tracking experiments were performed for each noise situation, and the total tracking time was plotted. As we can see from the result, when the entire FOV of the microscope was used as ROI (red line), the tracking time was drastically affected in a negative way by the increment in noise density. On the other hand, the effect of the noise density was reduced, and the tracking time was maintained by the implemented approach (green line) even in a highly noisy situation. In this experiment, only step 1, 2 of the visual tracking algorithm, explained in Section 2.4, are performed. In the second case, implemented approach, after step 2, the ROI was updated as explained in Section 2.4 (*a* = *b* = 20 in Equation (6), circle ROI) in a noiseless environment. The image noise was then generated after having a stable tracking situation. This result clearly shows the robustness of the implemented tracking algorithm, which has high potential to maintain the target tracking for a long time with low computational cost.

It is worth mentioning again that the vision sensor’s resolution and frame rate used in the previous experiments were 256 × 256 pixels at 500 FPS, respectively. In fact, the vision sensor can be operated at frame rates of up to 2000 FPS. However, based on the maximum speed of our target microorganism and the minimum size of the FOV, a frame rate of 500 FPS was confirmed to be suitable. Moreover, a frame rate higher that 500 FPS required a much stronger light source, which is not available in our platform. On the other hand, the maximum time required to change from the lowest magnification ratio (140×) to the highest magnification ratio (1400×) was approximately 1 s.

### 3.3. Application to Actual Motile Microorganism

For the actual motile microorganism experiment, *Paramecium* was chosen as a target. *Paramecium* is widely used in various studies as a model organism of eukaryotic cells and can be found in freshwater, including small ponds or lakes and other marine environments. *Paramecium* belongs to the ciliated group and it has an ovoid and elongated body shape with a body length of 100–200 µm. *Paramecia* basically propel themselves by whiplash movements of their cilia, giving it a movement speed of almost 1 mm/s. Interestingly, the swimming speed of *Paramecium* can reach up to 10 mm/s if it is stimulated; this is due to another actuator called trichocysts. Trichocysts are thin and long threads that are discharged from inside the body for a quick escape when the cell detects stimulation or danger. The undischarged trichocysts inside the *Paramecium* body are approximately 3–4 µm in length, and they elongate by 6–8 times when discharged [25]. Hamel et al. succeeded in stimulating immobilized *Paramecium* with heat stimulation using a focused laser beam [26]. Although they found several new gaits of the *Paramecium* by the trichocysts, the specific dynamic behavior of swimming *Paramecium* is still unknown.

For the experiment, wild-type *Paramecia* (*P. aurelia*) were prepared and cultivated in a medium with an incubator at 25 °C. One of the main motivations to use a microfluidic chip is to eliminate the occlusion between cells, without affecting their swimming mode as much as possible. This could be achieved by choosing the appropriate thickness of the chip. Therefore, we first fabricated microfluidic chips with a workspace thickness of 50, 100, 150, and 200 μm each. A small number of *Paramecia* were then introduced from the side aperture of the chip, and the behavior of the *Paramecia* was observed by the microscope. In the case of the 50-μm chip, the *Paramecium* frequently touched the upper and/or lower glass substrate, and the swimming mode was slightly changed. On the other hand, overlap of *Paramecia* occurred by a thick workspace with 150 and 200 μm. Therefore, we decided to use the 100-μm-thick microchip, which had no occlusion problem and did not have any noticeable effect on the *Paramecium* swimming mode.

**Figure 9:**
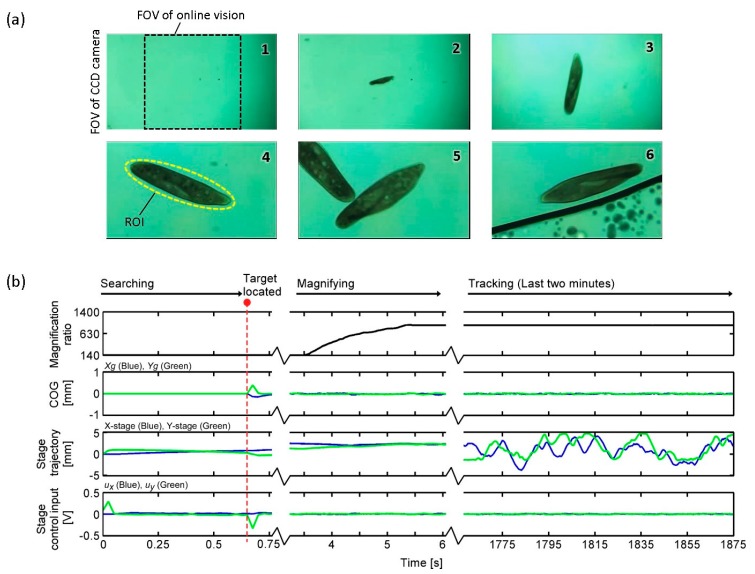
Results of long-time tracking of single *Paramecium* in a microfluidic chip. (**a**) Images of all tracking procedures; (**b**) Data log measured by the developed platform during the target tracking experiment (data sampling cycle was 10 ms).

**Figure 10:**
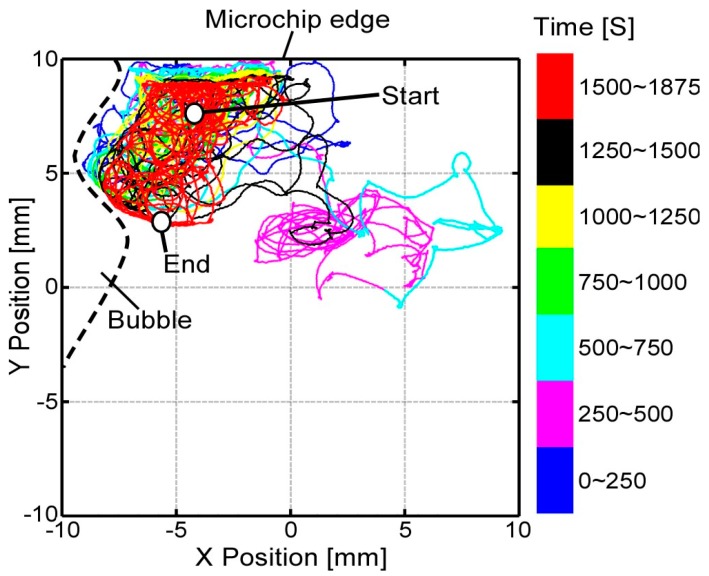
Swimming path of single *Paramecium* during 31 min and 15 s of target tracking. Maximum swimming speed of the *Paramecium* was approximately 2 mm/s.

Figure 9a,b shows the searching, tracking, and magnification procedures in the experiment. By considering the shape and size of the *Paramecium*, an elliptical ROI (*a* = 100, *b* = 20 in Equation (6)) with an 840× magnification ratio was used. The tracking was successfully conducted for more than 30 min regardless of image noises, such as microchip edge, debris, and other cells, as shown in Video S3. A total of four experiments were conducted for different *Paramecium*, and the tracking performance was 36 ± 5 min. Figure 10 shows an example of *Paramecium*’s swimming path during the experiment in Figure 9. The *Paramecium* position information were taken from the XY stage position sensors and the online vision data, where the mean value of the XY stage positioning error was approximately 40 µm. (Three other cases are shown in Figure S2.) From these experiments, we confirmed the effectiveness of the combined magnification control and visual tracking for efficient and long-time tracking.

**Figure 11:**
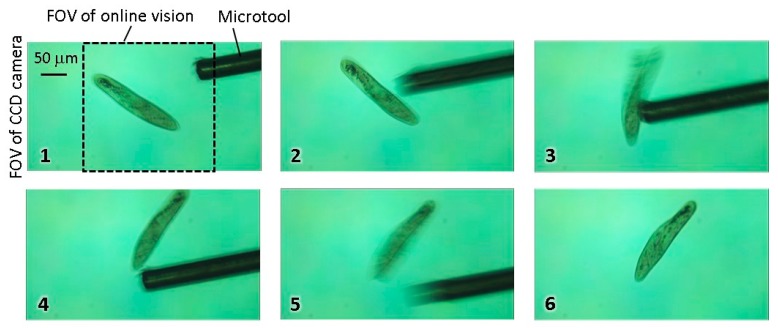
Experimental result for mechanical stimulation of swimming *Paramecium*.

Finally, a stimulation case was demonstrated to confirm the ability of the platform to continuously track the target while applying the mechanical stimulation. In this experiment, a φ50-μm hollowed microtool made of stainless steel was inserted from the side aperture of the microchip to apply the stimulation (an example of the fabricated microfluidic chip is shown in Figure S3.). Figure 11 and Video S4 show the experimental result. From this result, we confirmed that the tracking stability was maintained and that the mechanical stimulation can be applied in real time without destabilizing the COG calculation.

## 4. Discussion

One of the problems we faced while conducting the tracking experiment for a long time was the overheating of the XY stage, which forced us to stop the experiment after approximately 30 min to guarantee the platform’s safety. Because one of our main goals was to observe the lifespan of the microorganism in real time, particularly the cell division process, our platform should be able to track the microorganism for approximately one continuous day. Therefore, we would like to solve the heating problem in the future by adding external heatsinks or applying an air cooling mechanism to the stage in order to increase the overall tracking time. 

On the other hand, we utilized a 50-µm-thick microtool to apply mechanical stimulation. This is because using a thicker microtool would require increasing the microchip depth, resulting in more vertical movement of the microorganism and hence making the tracking process more difficult. In addition, the thicker the microtool, the stronger the generated fluidic disturbance when actuated in a microfluidic environment, because the viscosity of the fluid in the microspace is relatively high. These strong fluidic disturbances greatly affect the position of the target microorganism. Therefore, using a very thin microtool becomes essential. However, this kind of thin microtool tends to deflect easily when actuated inside of a fluid, which drastically reduces the tool positioning accuracy and makes the microorganism stimulation process more laborious with a low success ratio. Therefore, we would like to increase the positioning accuracy of the microtool by overcoming the generated deflection in future work.

Finally, after designing and implementing the stimulation system carefully and efficiently, our final goal would be to analyze the dynamic behavior of the microorganism. For instance, the input–output relationship of the response to the mechanical stimulation could be analyzed to obtain the structural and dynamical mathematical model of the microorganism, such as the transfer function model. This kind of knowledge can be very useful and inspiring in designing and fabricating novel robotic sensors and actuators.

## 5. Conclusions

A novel high-speed microrobotic platform that realizes long-time tracking and stimulation of a free motile microorganism in a microfluidic chip was demonstrated. The basic architecture and each component of the developed platform with a high-speed online vision sensor was explained. The four-step procedure for the motile microorganism investigation was also detailed. To realize real-time target tracking, the block diagram of the visual servo controller was shown, including the pixel pitch, focal length, and illumination control for the adaptive tracking. A simple image processing method, which utilizes the small spatial difference between two consecutive frames, was used, and its effectiveness against image noise was demonstrated. The platform could successfully track targets that move with a velocity of up to 10 mm/s even when using the largest magnification ratio available in the platform. Finally, the developed platform was used to conduct experiments using an actual motile microorganism. *Paramecium*, with average swimming speed of 1 mm/s, was chosen as a target. The platform could track the *Paramecium* continuously for more than 30 min and was successfully able apply mechanical stimulation with high magnification using a 50-µm-thick microtool inserted inside of the microfluidic chip without any immobilization process. We believe that by applying various kinds of stimulations to a freely swimming microorganism, we could initiate unknown reactions and behavior of the cell and lead to a better understanding of its gaits and further new findings. This developed platform could be an essential tool for the future investigation of motile microorganisms and can open the door for breakthrough discoveries in biological fields. 

## Supplementary Materials

The following are available online at www.mdpi.com/2072-666X/8/10/295/s1, Figure S1: Calibration results of three main parameters for magnification ratio control of microscope, Figure S2: Swimming path of single *Paramecium* in three different tracking experiments, Figure S3: An overview of fabricated microfluidic chip, Video S1: Real-time magnification ratio control of the microscope, Video S2: Basic experiments for the tracking performance evaluation, Video S3: Long-time tracking of single *Paramecium*, Video S4: Mechanical stimulation of *Paramecium* during the tracking.
