# Supplementary material for: Microrobotic Platform for Single Motile Microorganism Investigation
Source: Micromachines (Basel). 2017 Sep 30;8(10):295. doi: 10.3390/mi8100295 (PMC6189944; doi:10.3390/mi8100295)
Supplement: Supplementary file 1 [file micromachines-08-00295-s001.zip › Supplementary_Material.docx]

Microrobotic Platform for Single Motile Microorganism Investigation

Belal Ahmad ^1^, Hironobu Maeda ^2^, Tomohiro Kawahara ^1,^*, and Fumihito Arai ^3^


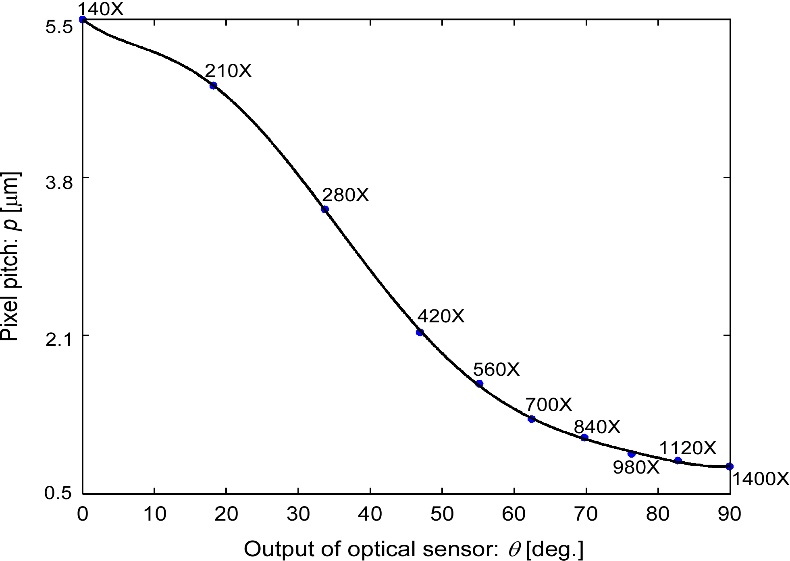


(a) *p*= *f*_1_(*θ*)=0.0001*θ* ^6^+0.0001*θ* ^5^-0.0006*θ ^4^*-0.00003*θ ^3^*+0.0014*θ* ^2^-0.0018*θ* +0.0017, *R*^2^*=0.99*


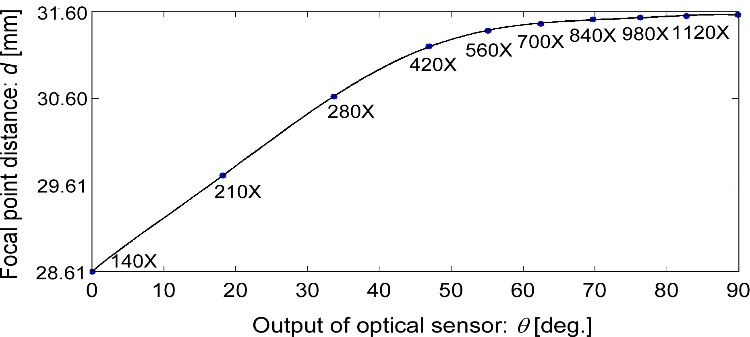


(b) *d*= *f*_2_(*θ*)=0.0001*θ* ^3^-0.0015*θ* ^2^+0.0702*θ* +28.6101, *R*^2^*=0.99*


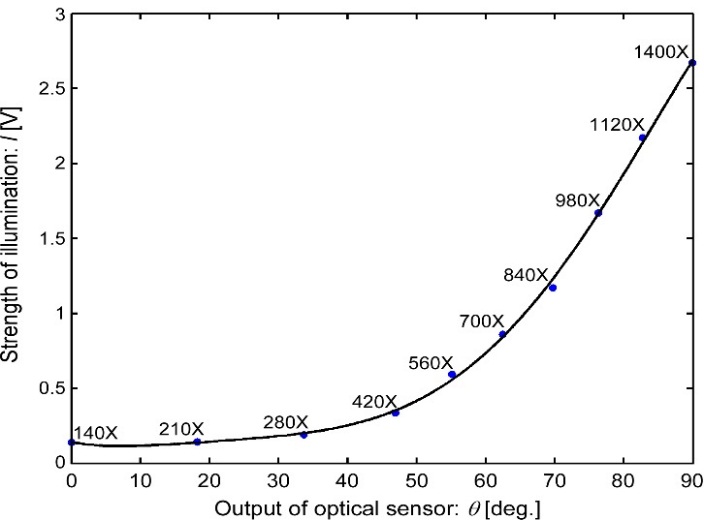


(c) *l*= *f*_3_(*θ*)=-0.0513*θ* ^5^-0.1058*θ ^4^*+0.1912*θ* ^3^+0.7398*θ* ^2^-0.8423*θ* +0.5083, *R*^2^*=0.99*

**Figure S1.** Calibration results of three main parameters for magnification ratio control of microscope. The function of *θ* (The dial rotational degree measured by the optical sensor) for the curve fitting and the coefficient of determination *R*^2^ are also shown. (**a**) Relationship between the magnification ratio and the pixel pitch of the captured image; (**b**) Relationship between the magnification ratio and the focal point distance of the microscope; (**c**) Relationship between the magnification ratio and the appropriate illumination strength of the microscope.


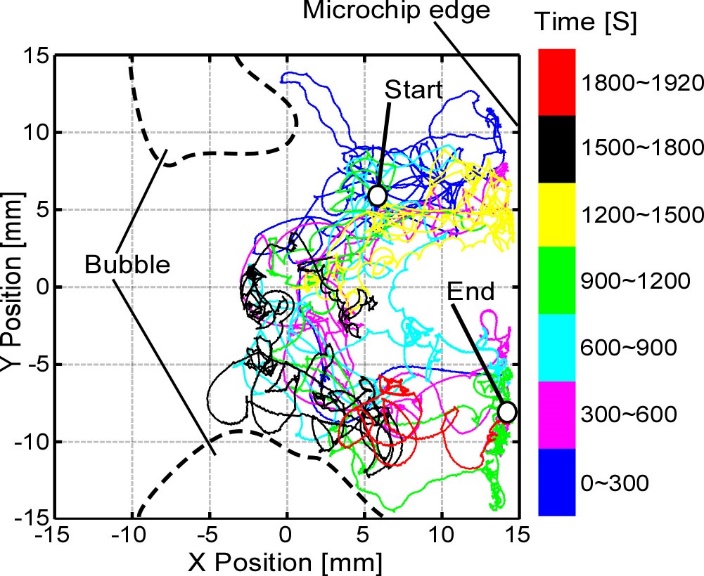


(a) Tracking duration: 32 min and 9 s, microchip dimensions: 30 × 30 × 0.1 mm


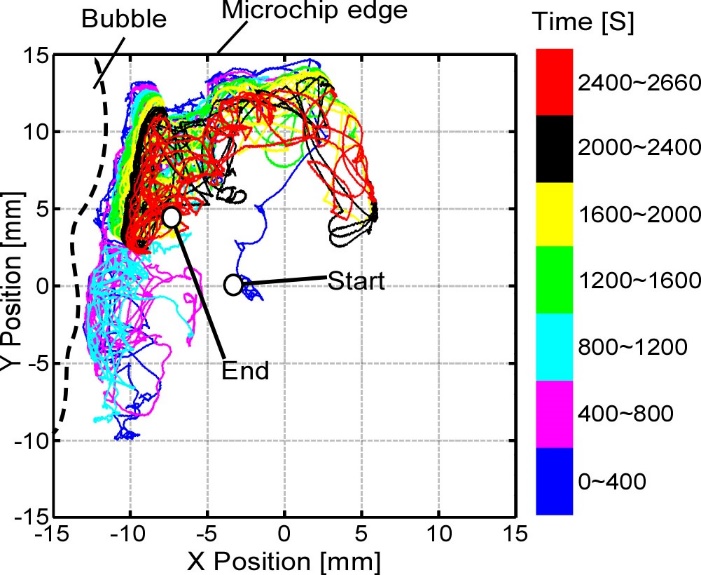


(b) Tracking duration: 44 min and 32 s, microchip dimensions: 30 × 30 × 0.1 mm


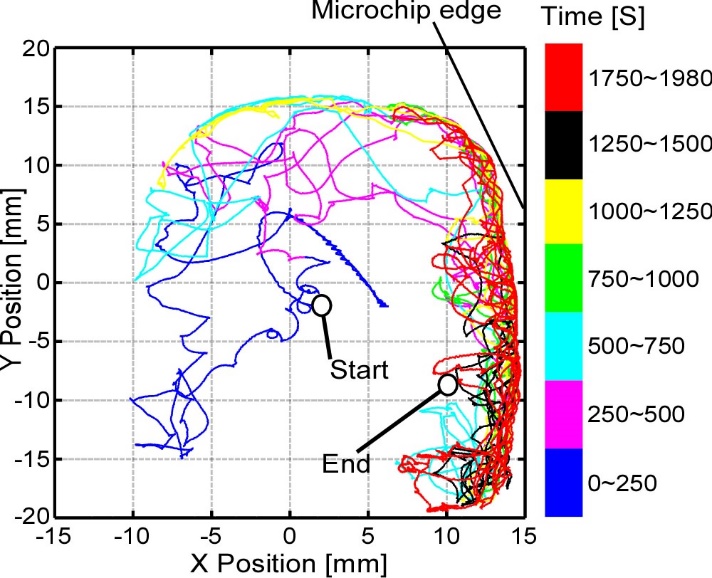


(c) Tracking duration: 33 min and 15 s, microchip dimensions: 30 × 40 × 0.1 mm

**Figure S2.** Swimming path of single *Paramecium* in three different tracking experiments.

**
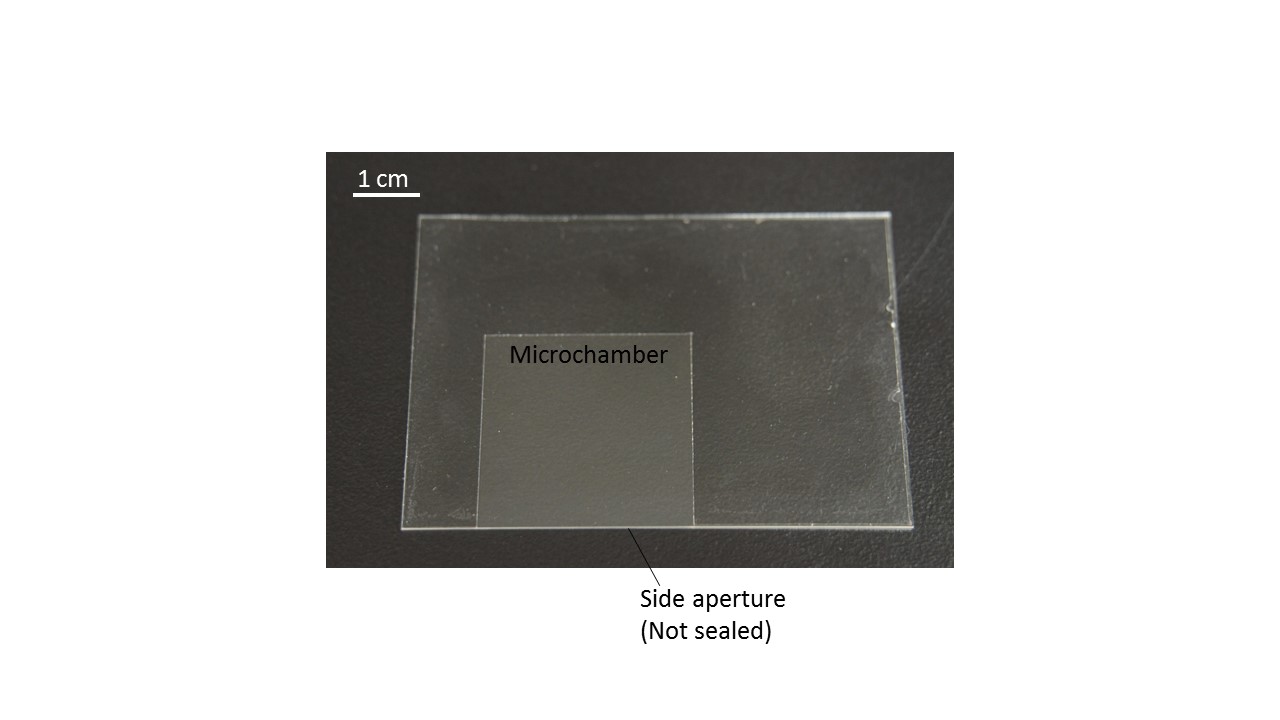
**

**Figure S3.** An overview of fabricated microfluidic chip. Chamber dimensions: 3 × 3 × 0.1 mm. The microtool can be inserted from the side aperture to apply stimulation.

© 2017 by the authors. Submitted for possible open access publication under the
terms and conditions of the Creative Commons Attribution (CC BY) license (http://creativecommons.org/licenses/by/4.0/).
